# Supplementary material for: FSH-Induced Nuclear Exclusion of FOXO1 Mediated by PI3K/Akt Signaling Pathway in Granulosa Cells Is Associated with Follicle Selection and Growth of the Hen Ovary
Source: Cells. 2025 Nov 26;14(23):1864. doi: 10.3390/cells14231864 (PMC12691273; doi:10.3390/cells14231864)
Supplement: Supplementary file 1 [file cells-14-01864-s001.zip › cells-3962413-supplementary.pdf]

Supplementary files:

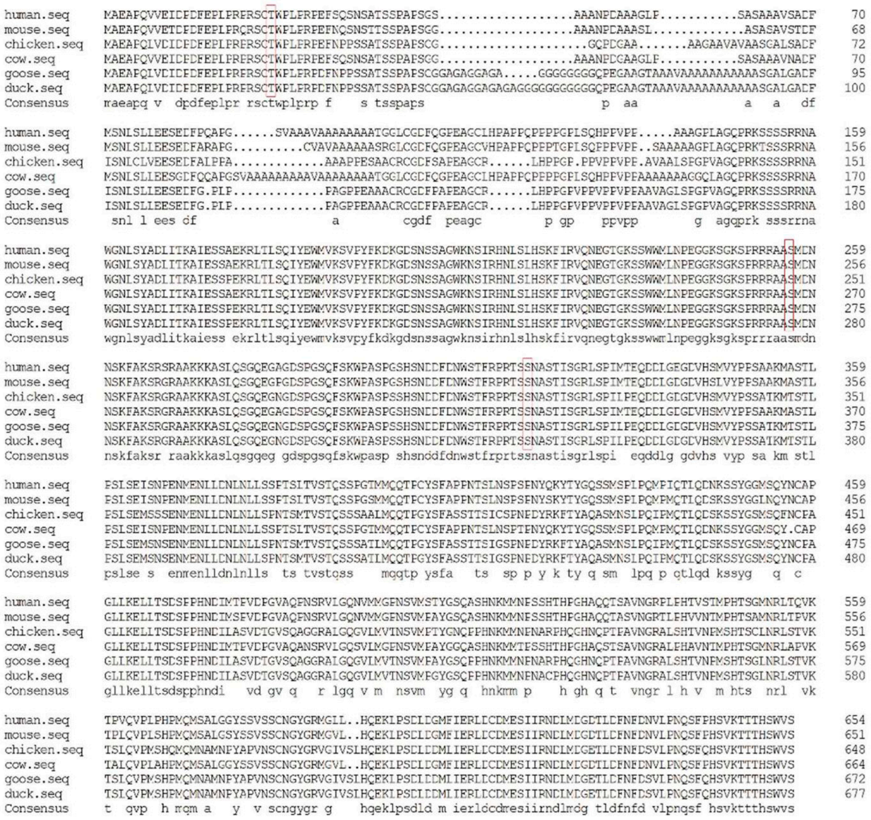

Supplemental Figure S1. Alignment of FOXO1 amino acid sequence of chicken with those of human, goose and duck. The conserved phosphorylation sites of each species were marked in red square.

```
Score            Expect Method                     Identities      Positives       Gaps
909 bits(2348)   0.0    Compositional matrix adjust. 499/660(76%)  549/660(83%)    16/660(2

Query  1    MAEAPQLVDIDPDFEPLPRPRSCTWPLPRPEFNPPSSATSSPAPSCGGQPDGAAAAGAAV  60
            MAEAPQ+V+IDPDFEPLPRPRSCTWPLPRPEF+  +SATSSPAPS  G       A A +
Sbjct  1    MAEAPQVVEIDPDFEPLPRPRSCTWPLPRPEFSQSNSATSSPAPS--GSAAANPDAAAGL  58

Query  61   AVAASGALSADFISNLCLVEESEDFALPPAAAAPPESAACR-------CGDFSAPEAGCR  113
              A++ A+SADF+SNL L+EESEDF   P + A   +AA         CGDF  PEAGC
Sbjct  59   PSASAAAVSADFMSNLSLLEESEDFPQAPGSVAAAVAAAAAAAATGGLCGDFQGPEAGC-  117

Query  114  LHPPGPPPVPPVP----PAVAALSPGPVAGQPRKSSSSRRNAWGNLSYADLITKAIESSP  169
            LHP  P P PP P    P V   + GP+AGQPRKSSSSRRNAWGNLSYADLITKAIESS
Sbjct  118  LHPAPPQPPPPGPLSQHPPVPPAAAGPLAGQPRKSSSSRRNAWGNLSYADLITKAIESSA  177

Query  170  EKRLTLSQIYEWMVKSVPYFKDKGDSNSSAGWKNSIRHNLSLHSKFIRVQNEGTGKSSWW  229
            EKRLTLSQIYEWMVKSVPYFKDKGDSNSSAGWKNSIRHNLSLHSKFIRVQNEGTGKSSWW
Sbjct  178  EKRLTLSQIYEWMVKSVPYFKDKGDSNSSAGWKNSIRHNLSLHSKFIRVQNEGTGKSSWW  237

Query  230  MLNPEGGKSGKSPRRRAASMDNNSKFAKSRGRAAKKKASLQSGQEGNGDSPGSQFSKWPA  289
            MLNPEGGKSGKSPRRRAASMDNNSKFAKSR RAAKKKASLQSGQEG GDSPGSQFSKWPA
Sbjct  238  MLNPEGGKSGKSPRRRAASMDNNSKFAKSRSRAAKKKASLQSGQEGAGDSPGSQFSKWPA  297

Query  290  SPSSHSNDDFDNWSTFRPRTSSNASTISGRLSPILPEQDDLGDGDVHSMVYPSSATKMTS  349
            SP SHSNDDFDNWSTFRPRTSSNASTISGRLSPI+ EQDDLG+GDVHSMVYP SA KM S
Sbjct  298  SPGSHSNDDFDNWSTFRPRTSSNASTISGRLSPIMTEQDDLGEGDVHSMVYPPSAAKMAS  357

Query  350  TLPSLSEMSSSENMENLLDNLNLLSPNTSMTVSTQSSSAALMQQTPGYSFASSTTSICSP  409
            TLPSLSE+S+ ENMENLLDNLNLLS  TS+TVSTQSS   +MQQTP YSFA   TS+ SP
Sbjct  358  TLPSLSEISNPENMENLLDNLNLLSSPTSLTVSTQSSPGTMMQQTPCYSFAPPNTSLNSP  417

Query  410  NPDYRKFTYAQASMNSLPQIPMQTLQDSKSSYGSMSQFNCPAGLLKELLTSDSPPHNDIL  469
            +P+Y+K+TY Q+SM+ LPQ+P+QTLQD+KSSYG MSQ+NC  GLLKELLTSDSPPHNDI+
Sbjct  418  SPNYQKYTYGQSSMSPLPQMPIQTLQDNKSSYGGMSQYNCAPGLLKELLTSDSPPHNDIM  477

Query  470  ASVDTGVSQAGGRALGQGVLMVTNSVMPTYGNQPPHNKMMNPNARPHQGHNQPTPAVNGR  529
              VD GV+Q   R LGQ V+M  NSVM TYG+Q  HNKMMNP++  H GH Q T AVNGR
Sbjct  478  TPVDPGVAQPNSRVLGQNVMMGPNSVMSTYGSQASHNKMMNPSSHTHPGHAQQTSAVNGR  537

Query  530  ALSHTVNPMSHTSCLNRLSTVKTSLQVPMSHQMQMNAMNPYAPVNSCNGYGRVGIVSLHQ  589
             L HTV+ M HTS +NRL+ VKT +QVP+ H MQM+A+  Y+ V+SCNGYGR+G+  LHQ
Sbjct  538  PLPHTVSTMPHTSGMNRLTQVKTPVQVPLPHPMQMSALGGYSSVSSCNGYGRMGL--LHQ  595

Query  590  EKLPSDLDDMLIERLDCDMESIIRNDLMDGETLDFNFDSVLPNQSFQHSVKTTTHSWVSG  649
            EKLPSDLD M IERLDCDMESIIRNDLMDG+TLDFNFD+VLPNQSF HSVKTTTHSWVSG
Sbjct  596  EKLPSDLDGMFIERLDCDMESIIRNDLMDGDTLDFNFDNVLPNQSFPHSVKTTTHSWVSG  655
```

Supplemental Figure S2. Alignment of FOXO1 amino acid sequence of chicken with that of human. The protein amino acid sequence of chicken lies on the top line and that of human lies under. The yellow sequence denotes NLS sequence. The FOXO1 Ser[248]/Ser[256] site was marked in red square.

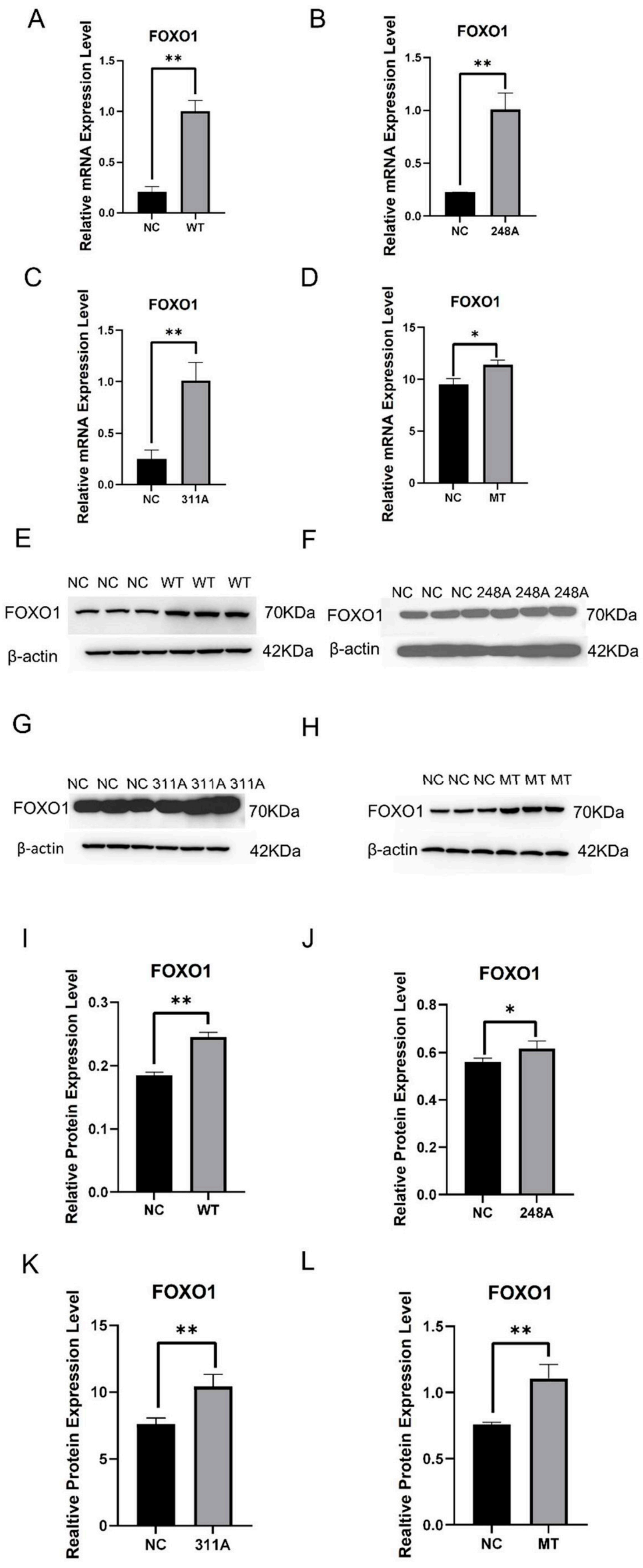
A
FOXO1
Relative mRNA Expression Level
**
NC
WT
B
FOXO1
Relative mRNA Expression Level
**
NC
248A
C
FOXO1
Relative mRNA Expression Level
**
NC
311A
D
FOXO1
Relative mRNA Expression Level
*
NC
MT
E
NC NC NC WT WT WT
FOXO1 70KDa
β-actin 42KDa
F
NC NC NC 248A 248A 248A
FOXO1 70KDa
β-actin 42KDa
G
NC NC NC 311A 311A 311A
FOXO1 70KDa
β-actin 42KDa
H
NC NC NC MT MT MT
FOXO1 70KDa
β-actin 42KDa
I
FOXO1
Relative Protein Expression Level
**
NC
WT
J
FOXO1
Relative Protein Expression Level
*
NC
248A
K
FOXO1
Realtive Protein Expression Level
**
NC
311A
L
FOXO1
Realtive Protein Expression Level
**
NC
MT

Supplemental Figure S3. Overexpression efficiency of four types of FOXO1 overexpression vectors. (A) mRNA overexpression efficiency of FOXO1 wild type overexpression vector was determined by qPCR analysis. (B)FOXO1 Ser248 mutant overexpression vector was determined by qPCR analysis. (C) FOXO1 Ser311 mutant overexpression vector was determined by qPCR analysis. (D) FOXO1 MT mutant overexpression vector was determined by qPCR analysis. (E) The protein expression of FOXO1 in the cultured GCs under the FOXO1 wild type overexpression were determined via western blotting by using the anti-FOXO1 respectively. (F) The protein expression of FOXO1 in the cultured GCs under the FOXO1 Ser248A overexpression were determined via western blotting by using the anti-FOXO1 respectively. (G) The protein expression of FOXO1 in the cultured GCs under the FOXO1 Ser311A overexpression were determined via western blotting by using the anti-FOXO1 respectively. (H) The protein expression of FOXO1 in the cultured GCs under the FOXO1 Ser311A overexpression was determined via western blotting by using the anti-FOXO1 respectively. β-actin was used as a loading control. All blots were cropped, and the gels were run under the same experimental conditions. (I) The result of FOXO1 protein expression level under FOXO1 wild type overexpression by using western blotting. (J) The result of FOXO1 protein expression level under FOXO1 Ser248A overexpression by using western blotting. (K) The result of FOXO1 protein expression level under FOXO1 Ser311A overexpression by using western blotting. (K) The result of FOXO1 protein expression level under FOXO1 MT overexpression by using western blotting. * and ** denote $P < 0.05$ and $P < 0.01$, respectively. All data are presented as the means ± SEM. n= 5.

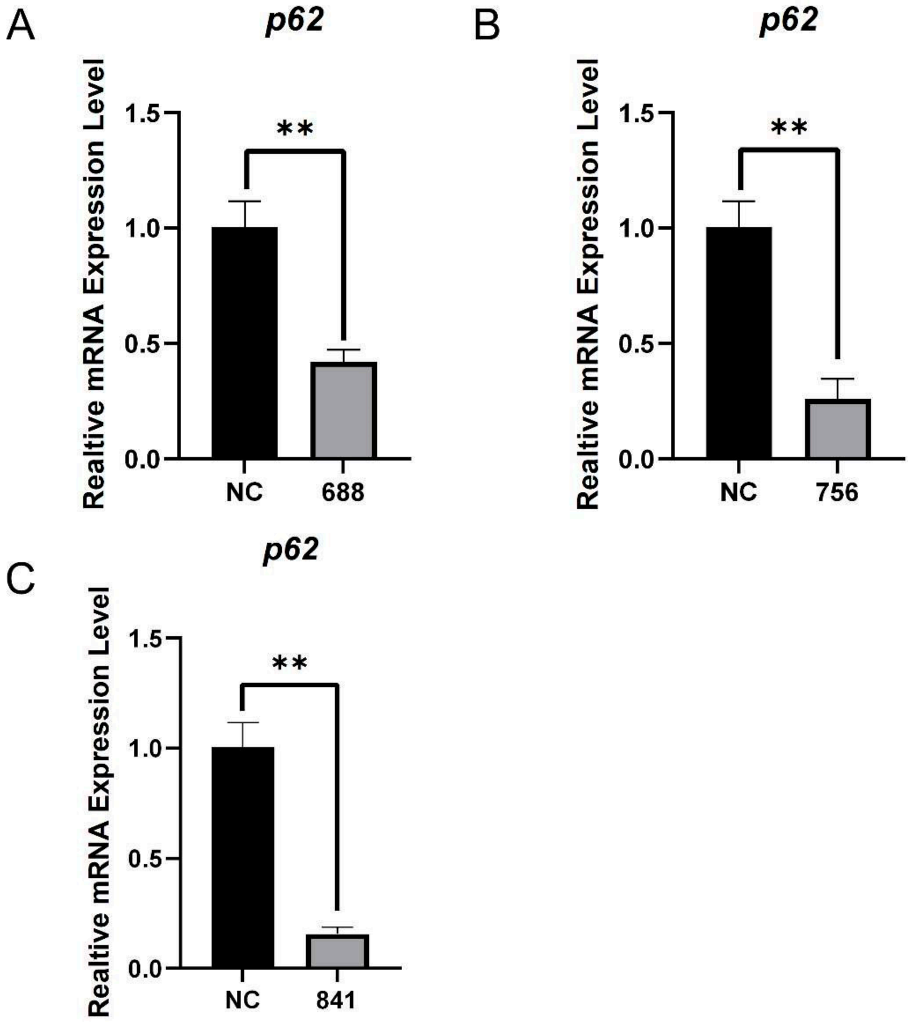

Supplemental Figure S4. Interference efficiency of siRNA on p62, (A) Interference efficiency of RNAi 688 was determined by qPCR analysis. (B) Interference efficiency of RNAi 756 was determined by qPCR analysis. (C) Interference efficiency of RNAi 841 was determined by qPCR analysis. The relative expression level was expressed as fold change over the control. *18S RNA* gene was used as the internal control gene. ** denotes $P < 0.01$. All data are presented as the means ± SEM. n= 5.
